# ANO1/TMEM16A regulates process maturation in radial glial cells in the developing brain

**DOI:** 10.1073/pnas.1901067116

**Published:** 2019-05-30

**Authors:** Gyu-Sang Hong, Sung Hoon Lee, Byeongjun Lee, Jae Hyouk Choi, Soo-Jin Oh, Yongwoo Jang, Eun Mi Hwang, Hyungsup Kim, Jooyoung Jung, In-Beom Kim, Uhtaek Oh

**Affiliations:** ^a^Brain Science Institute, Korea Institute of Science and Technology, 02792 Seoul, Korea;; ^b^College of Pharmacy, Chung-ang University, 06974 Seoul, Korea;; ^c^Division of Bio-Medical Science and Technology, Korea Institute of Science and Technology, 02792 Seoul, Korea;; ^d^Department of Biomedical Engineering, Hanyang University, 04763 Seoul, Korea;; ^e^Department of Anatomy, College of Medicine, Catholic University, 06591 Seoul, Korea

**Keywords:** Anoctamin 1, TMEM16A, neural stem cell, radial glial cell, cortical development

## Abstract

Neural stem cells (NSCs) are primary progenitor cells in the early developmental stage in the brain that initiate a diverse lineage of differentiated neurons and glia. Radial glial cells (RGCs), a type of neural stem cell in the ventricular zone, are essential for nurturing and delivering new immature neurons to the appropriate cortical target layers. Here we report that Anoctamin 1 (ANO1)/TMEM16A, a Ca^2+^-activated chloride channel, mediates the Ca^2+^-dependent process extension of RGCs. ANO1 is highly expressed and functionally active in RGCs of the mouse embryonic ventricular zone. Knockdown of ANO1 suppresses RGC process extension and protrusions, whereas ANO1 overexpression stimulates process extension. Among various trophic factors, brain-derived neurotrophic factor (BDNF) activates ANO1, which is required for BDNF-induced process extension in RGCs. More importantly, *Ano1*-deficient mice exhibited disrupted cortical layers and reduced cortical thickness. We thus conclude that the regulation of RGC process extension by ANO1 contributes to the normal formation of mouse embryonic brain.

## Significance

Radial glial cells (RGCs), a type of neural stem cell in the developing brain, not only generate progenitors, newly born neurons and glial cells, but also deliver neurons through its process to the appropriate cortical target layers. Thus, the function of RGCs is crucial for cortex development, in which Cl^−^ channels are thought to play a role. Here we highlight that Anoctamin 1 (ANO1)/TMEM16A, a Ca^2+^-activated Cl^−^ channel, mediates the process extension in RGCs. ANO1-null mice show a decrease in cortical thickness with disorganized cortical layers. Thus, as a Cl^−^ channel, ANO1 is involved in the process maturation of RGCs and contributes to cortex development.

Development of the mammalian central nervous system requires cell division for neurogenesis and gliogenesis through a diverse lineage of neural stem cells (NSCs) (1). In addition, neuronal migration to the correct final destinations is required, in which immature neurons differentiate into mature neurons to form cortical layers (2, 3).

Radial glial cells (RGCs), a specialized subpopulation of NSCs, are generated from neuroepithelial cells that form a neural plate during neocortical development (1). RGCs not only are symmetrically divided into new RGCs, but also directly differentiate into immature neurons or indirectly into intermediate progenitor cells that further mature into immature neurons. In early embryonic brain development, the ventricular zone (VZ) is formed near the ventricle. Above the VZ, a subventricular zone (SVZ) emerges, in which a variety of proliferative processes occur (1, 3).

Although the involvement of neurogenesis or gliogenesis differs across species, RGCs of different species share common critical features, such as a proliferative function and guidance of immature neurons (1). The RGC somas are localized in the VZ or SVZ in the embryonic brain. Typical RGCs are bipolar, with a long pial-directed process and a short process in contact with the surface of the ventricle. The long processes of RGCs guide newborn immature neurons to their target laminar layers. Thereafter, an “inside-out” laminar arrangement of six cortical layers develops from layer VI to layer I (1). After delivering immature neurons, RGCs settle into proper cortical layers, where their processes retract. Thereafter, RGCs remain as astroglia that further induce adult neurogenesis (4). In primate developing cortex, other types of RGCs, such as highly proliferative outer RGCs with a monopolar shape, produce large numbers of neural progenitor cells in the outer SVZ (2, 5). These outer RGCs act as a scaffold for the cortical architecture and contribute to gyrification of the cortex in primates. Because these outer RGCs are largely lacking in rodents, the cortical folds are less prominent than those in primates (2, 3, 6). Thus, the proper development of RGCs, such as accurate process extension, is essential for normal cortical development (1, 2).

During embryonic brain development, ion channels are responsible for the direct or indirect physiological functions of NSCs. Among these, gamma-aminobutyric acid A (GABA_A_) receptors induce the depolarization of NSCs (7). A higher intracellular Cl^−^ concentration in neurons in the embryonic brain results in the depolarization induced by GABA_A_ receptors. The depolarized membrane potential activates voltage-gated Ca^2+^ channels, resulting in an increase in Ca^2+^ transients, which eventually control proliferation, neuronal migration, and process elongation (8–10). Although the depolarization by Cl^−^ efflux is essential for NSC physiology, the role of chloride channels other than GABA_A_ receptors in NSCs during cortical development remains elusive.

Anoctamin 1 (ANO1, or TMEM16A) is a Cl^−^ channel activated by intracellular Ca^2+^ that is expressed in many organs (11–13). Recent cryo-EM analyses revealed that ANO1 forms a dimer with two pore regions (14, 15). The Anoctamin gene family has 10 isoforms, ANO1–ANO10; however, only ANO1 and ANO2 (TMEM16B) are known to conduct Ca^2+^-activated chloride currents under physiological conditions (11, 16). ANO1 is involved in the regulation of epithelial fluid secretion, cardiac excitability, and smooth muscle contraction and nociception (17). ANO1 is highly expressed in various tumor cells (18). However, its role in the brain is largely unknown, owing to its absence in the mature brain (16, 19). Interestingly, *Ano1* transcripts are expressed in the ventricular neuroepithelium during brain development (20), suggesting that ANO1 plays a role in neural development. Because the Cl^−^ efflux-induced depolarization is essential for NSC function, a role for ANO1 in the embryonic brain is expected. Thus, the present study aimed to determine the role of ANO1 in brain development.

## Results

### ANO1 Is Highly Expressed in Embryonic Cortices.

**Fig. 1.**
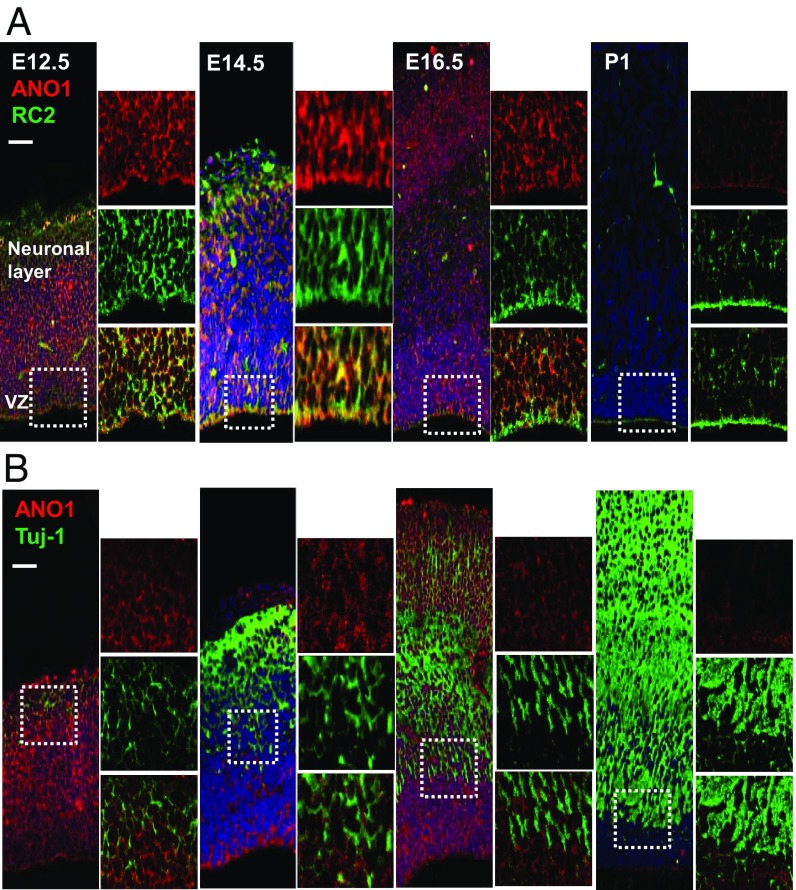
ANO1 expression in the developing mouse brain. (*A* and *B*) Immunofluorescence images of mouse brains at E12.5, E14.5, E16.5, and P1. Each brain section was stained with ANO1 and RC2 (*A*) or with ANO1 and Tuj-1 (*B*) antibodies. The small images on the *Right*-hand panels show magnification of the boxed region. (Scale bar: 50 μm.)

To validate the specific location of ANO1 in the developing brain, we performed immunostaining on embryonic mouse brain with the ANO1 antibody at embryonic day (E) 12.5, E14.5, E16.5, and postnatal day (P) 1. In the embryonic brain, ANO1 was highly expressed in the VZ and SVZ at E12.5 and E14.5, but expression was dramatically decreased at P1 (Fig. 1*A*). ANO1 was colocalized with RC2, an RGC marker, but failed to colocalize with neuron-specific class III p-tubulin (Tuj-1), a neuronal marker. Tuj-1 immunofluorescence was low at E12.5 but gradually increased from E14.5 to P1 (Fig. 1*B*). These results suggest that ANO1 is expressed in RGCs in the VZ and SVZ regions but not in neurons of the neonatal brain.

### ANO1 Is Functional in RGCs.

We further investigated whether ANO1 is functional in cultured RGCs. These RGCs were obtained from neurospheres prepared from mouse embryonic cortices. A subpopulation of these cells showed oval soma with short and long processes, a typical bipolar RGC morphology (*SI Appendix*, Fig. S1*A*) (21). These cells expressed ANO1 and RC2 (*SI Appendix*, Fig. S1*A*). Moreover, ANO1 and nestin, a marker of NSC, were expressed at day in vitro (DIV) 3 but showed decreased expression at DIV8 during in vitro differentiation, whereas neuronal and glial markers such as Tuj-1, glutamate decarboxylase 67, synapsin 1, and glial fibrillary acidic protein, showed higher expression levels at DIV8 (*SI Appendix*, Fig. S1*B*).

To test whether ANO1 is functional in RGCs, we measured Ca^2+^-activated Cl^−^ currents in cultured RGCs. To measure the ANO1-dependent Cl^−^ currents, 140 mM of CsCl and *N*-methyl-d-glucamine-Cl were used for pipette and bath solutions, respectively. In the whole-cell configuration with a holding potential of −80 mV, 1 μM of Ca^2+^ in the pipette induced robust Cl^−^ currents, which were not observed in RGCs transfected with *Ano1* siRNA (*SI Appendix*, Fig. S1*C*). The knockdown efficiency of ANO1 in the cultured RGCs was confirmed by RT-PCR and Western blot analysis (*SI Appendix*, Fig. S2 *A* and *B*). Voltage steps applied to the cultured RGCs evoked slowly activating currents, typical voltage-activated ANO1 currents (*SI Appendix*, Fig. S1*D*) (11). The current-voltage relationship in RGCs was outwardly rectifying, another indication of ANO1 current (11). The voltage-activated Cl^−^ currents were greatly reduced in *Ano1* siRNA-transfected RGCs (*SI Appendix*, Fig. S1*D*). In cultured RGCs, transcripts of *Ano3*, *Ano4*, and *Ano10* were also found along with those of *Ano1*; however, transcripts of *Ano2*, another Ca^2+^-activated Cl^−^ channel in the Anoctamin gene family, were rarely detected (*SI Appendix*, Fig. S2*C*). These results suggest that ANO1 may be one of functional Ca^2+^-activated Cl^−^ channels in RGCs.

### *Ano1* Down-Regulation Suppresses Process Extension in RGCs.

**Fig. 2.**
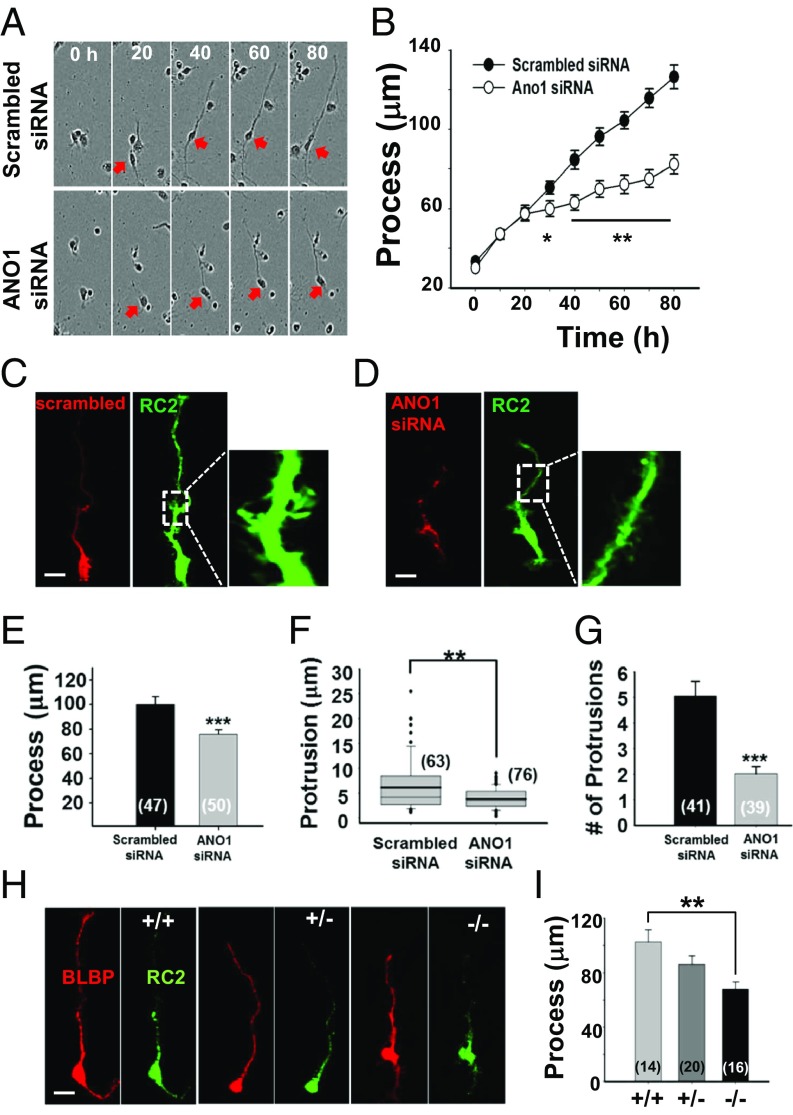
Inhibition of the RGC process extension by *Ano1* disruption. (*A*) The length of RGC processes was monitored in a time-lapse imaging chamber. RGCs transfected with scrambled or *Ano1* siRNA were cultured for 80 h. Representative images show cultured RGCs with process extension at given time points. Cell somas are marked with red arrows. (*B*) Summary of the length of RGC processes at given time points. *P < 0.05, **P < 0.01, Student’s *t* test (*n* = 20). (*C* and *D*) Representative images of processes of cultured RGCs. RGCs were transfected with Cy3-tagged scrambled siRNA (*C*) or *Ano1* siRNA (*D*). RGCs were also stained with RC2 (green) antibody for better visualization of their processes. (*Right*) Expanded region of the white dashed lines. (Scale bar: 20 μm.) (*E*–*G*) Summary of the process length (*E*), protrusion length (*F*), and number of protrusions (*G*) per 50 μm. All experiments were validated from three to five independent cultures. Numbers in the bracket represent the number of processes or protrusions analyzed. ***P < 0.001, Student’s *t* test. (*H*) Representative images of RGCs isolated from cortices of *Ano1*^*+/+*^, *Ano1*^*+/−*^, and *Ano1*^*−/−*^ mice at E12.5. Isolated cells were immunostained with BLBP and RC2. (Scale bar: 20 μm.) (*I*) Summary of the process lengths of BLBP^+^ and RC2^+^ cells isolated from each genotype. **P < 0.01, one-way ANOVA and Tukey’s post hoc test.

To investigate whether the decreased expression of ANO1 affects RGC development in vitro, we monitored cultured RGCs in a live-cell imaging chamber for 80 h after transfection of cy3-tagged scrambled or *Ano1* siRNA. We found that the RGCs transfected with scrambled siRNA extended their processes at ∼1.25 μm/h, whereas those transfected with *Ano1* siRNA showed extension at ∼0.63 μm/h (Fig. 2 *A* and *B*). Thus, the process length of RGCs was markedly reduced after *Ano1* siRNA transfection.

We next measured and counted the number and length of protrusions in each RGC process after staining with RC2 antibody (Fig. 2 *C* and *D*). Consistent with the live-cell imaging, we confirmed dramatically decreased process length in *Ano1* siRNA-treated RGCs (Fig. 2*E*). The mean length of protrusions in processes of scrambled siRNA-treated RGCs was 6.2 ± 0.7 μm (*n* = 63 protrusions from 15 cells) at DIV3, significantly longer than that in processes of RGCs transfected with *Ano1* siRNA (3.9 ± 0.2 μm; *n* = 76 protrusions from 25 cells; *P* < 0.01, Student’s *t* test) (Fig. 2*F*). The mean number of protrusions within 50 μm of processes was significantly lower in RGCs transfected with *Ano1* siRNA (5.1 ± 0.6 μm, *n* = 41 cells) compared with scrambled siRNA-treated RGCs (2.0 ± 0.3 μm, *n* = 39 cells; *P* < 0.001, Student’s *t* test) (Fig. 2*G*).

We next determined whether the genetic ablation of *Ano1* also suppresses RGC process extension. RGCs were directly isolated from embryonic cortices of littermate wild-type (*Ano1*^+/+^), heterozygote (*Ano1*^+/−^), and *Ano1* knockout (*Ano1*^−/−^) mice at E12.5. Most of these cells were positive for RC2 and brain lipid-binding protein (BLBP), another marker of RGCs (Fig. 2*H*). Consistent with the results of *Ano1* siRNA treatment, *Ano1*^−/−^ RGCs exhibited significantly shorter processes compared with *Ano1*^+/+^ RGCs (*Ano1*^+/+^, 102.6 ± 8.9; *Ano1*^+/−^, 86.1 ± 6.2; *Ano1*^−/−^, 67.9 ± 5.3 μm; *n* = 14, 20, and 16 cells, respectively; *P* < 0.01 *Ano1*^−/−^ vs. *Ano1*^+/+^, one-way ANOVA and Tukey’s post hoc test) (Fig. 2*I*). These results suggest that ANO1 mediates RGC process maturation.

### Up-Regulation of *Ano1* Stimulates RGC Process Extension.

**Fig. 3.**
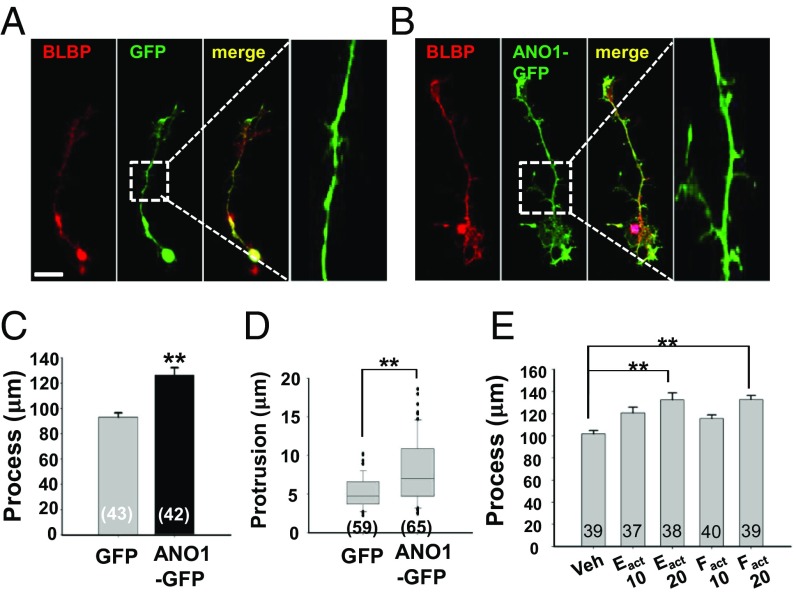
The process extension in RGCs after transfection or pharmacologic activation of *Ano1*. (*A* and *B*) Representative immunofluorescence images of RGCs transfected with *Gfp* (*A*) or *Ano1-Gfp* (*B*). (*Right*) Expanded images of the regions in white squares. (Scale bar: 20 μm.) (*C* and *D*) Summary of the lengths of processes (*C*) and protrusions (*D*) of RGCs transfected with *Gfp* or *Ano1-Gfp*. All experiments involving counting were validated from three to five independent cultures. (*E*) Summary of the process length of cultured RGCs pretreated with E_act_ (10 or 20 μM) and F_act_ (10 or 20 μM) for 3 d. RGCs were immunostained with BLBP to measure process length. **P < 0.01, one-way ANOVA and Tukey’s post hoc test.

Next, we tested whether overexpression of ANO1 in RGCs affects the extension and protrusions of RGC processes. To express ANO1 in RGCs, *Ano1-Gfp* or *Gfp* was transfected to cultured RGCs derived from neurospheres. The length of processes (*Gfp* vs. *Ano1-Gfp*: 93.7 ± 3.3 vs. 126.3 ± 5.9 μm; *n* = 43 and 42, respectively, *P* < 0.01; Student’s *t* test) and the length of protrusions (*Gfp* vs. *Ano1-Gfp*, 5.2 ± 0.3 vs. 7.9 ± 0.5; *n* = 59 protrusions from 18 cells and 65 protrusions from 20 cells, respectively; *P* < 0.01, Student’s *t* test) were also significantly increased in ANO1-GFP– expressing cells compared with GFP-expressing cells (Fig. 3 *A*–*D*). Similarly, when E_act_ and F_act_, specific activators of ANO1 (22), were applied to the cultured RGCs, the processes of cultured RGCs were markedly elongated (Fig. 3*E*).

### ANO1 Is Functional in Embryonic Brain Slices.

**Fig. 4.**
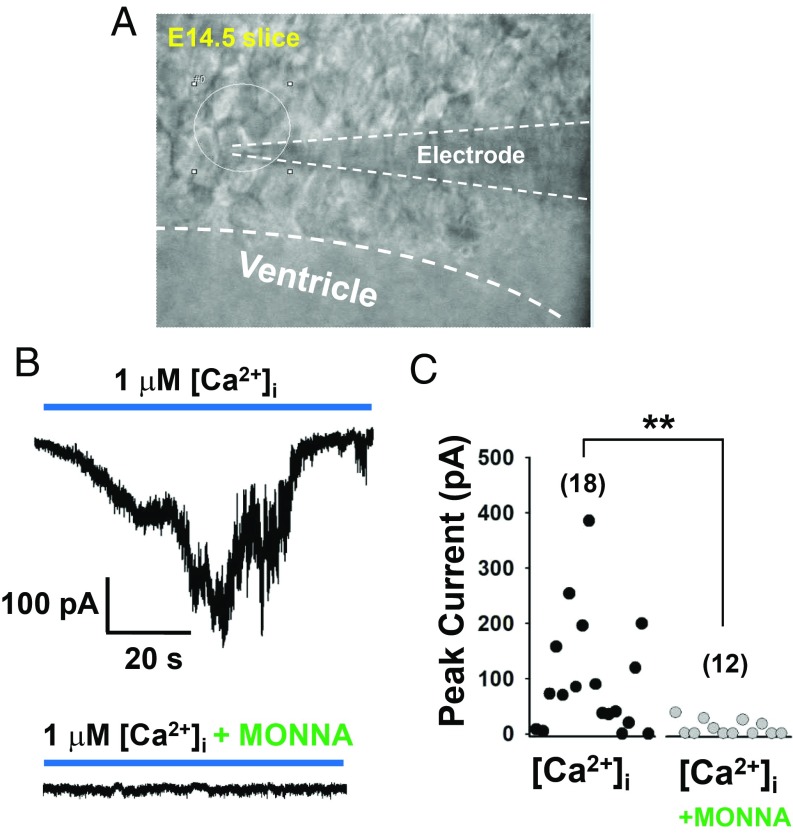
ANO1 is functional in embryonic brain slices. (*A*) A captured image of a cultured RGC in an E14.5 embryonic brain slice in which whole-cell currents were recorded. The recording glass electrode is marked by dashed lines. (*B*) Representative traces of Ca^2+^–induced Cl^−^ currents in RGCs in embryonic brain slices with or without 100 μM MONNA, an ANO1-specific blocker. To record Ca^2+^-activated Cl^−^ currents, the pipette contained 1 μM Ca^2+^ in 140 mM CsCl solution. Oxygenated artificial cerebrospinal fluid was used for the bath solution; *E*_hold_ = −70 mV. (*C*) Summary of the amplitudes of Ca^2+^-activated Cl^−^ currents in RGCs in embryonic brain slices with or without MONNA.

To test whether ANO1 in RGCs is also functional in the embryonic brain, we recorded Cl^−^ currents activated by intracellular Ca^2+^ in RGCs of E14.5 brain slices. Cells with a typical long process in the VZ were selected for RGCs (Fig. 4*A*). In the whole-cell configuration with the holding potential of −70 mV, intracellular 1 μM Ca^2+^ (in the pipette) induced robust Cl^−^ currents >50 pA in 14 out of 18 RGCs (mean value, 98.6 ± 24.6 pA; *n* = 18, four mice). However, when we pretreated the brain slices with 100 μM MONNA, a specific ANO1 blocker (23), the average amplitudes of cells with Cl^−^ currents were dramatically decreased (7.8 ± 3.1 pA; *n* = 12, three mice) (Fig. 4 *B* and *C*). Treated with MONNA, only 4 out of 14 cells showed Cl^−^ currents >50 pA. Thus, ANO1 appears to be functional in RGCs of embryonic brain.

### *Ano1* Ablation Disrupts the Cortical Organization.

**Fig. 5.**
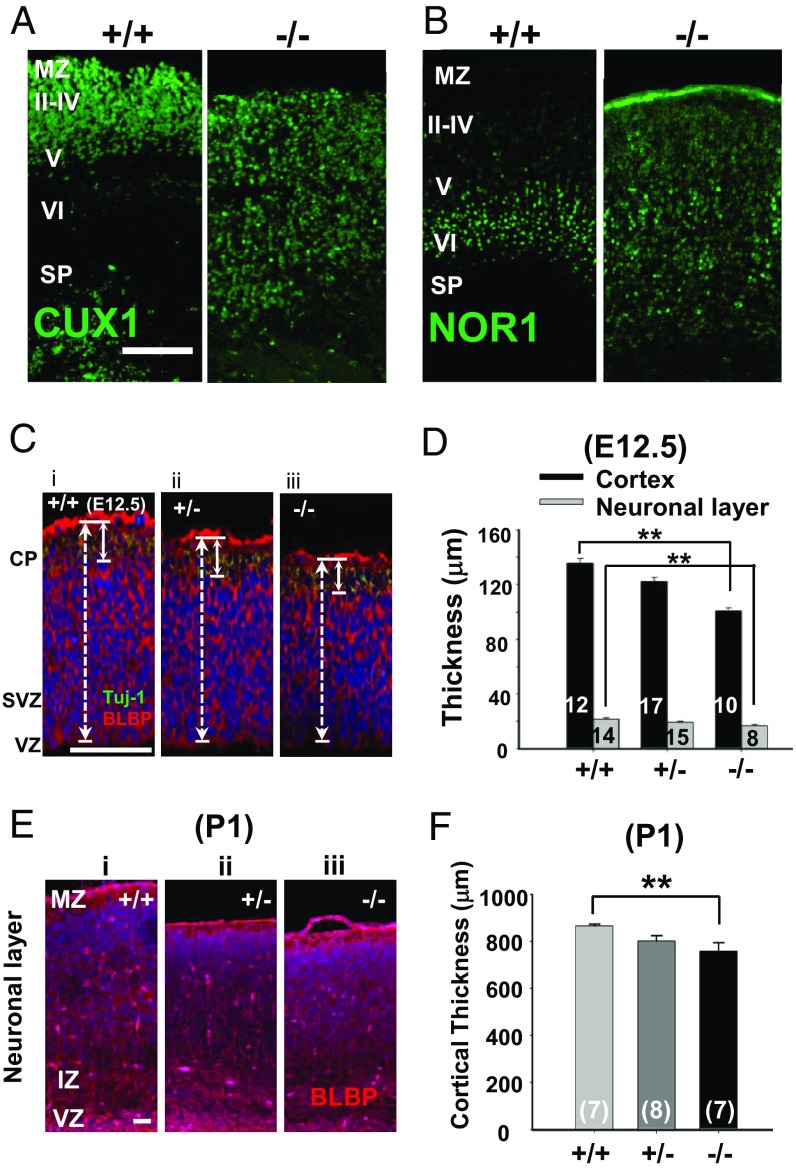
Layer organization was disrupted in the *Ano1*^*−/−*^ neonatal cortex. (*A* and *B*) Cortices of neonatal *Ano1*^+/+^ and *Ano1*^−/−^ mouse brains (P1) were stained with CUX1 (*A*) or NOR1 (*B*), markers for layers II–IV and layers V and VI, respectively. Note that layer specific clusters of CUX1^+^ or NOR1^+^ neurons in the specific layers of the cortex are absent in *Ano1*^*−/−*^ neonates. (Scale bar: 100 μm.) MZ, marginal zone; SP, subplate. (*C* and *E*) Representative images of *Ano1*^+/+^, *Ano1*^+/−^, and *Ano1*^−/−^ E12.5 (*C*) and P1 (*E*) cortices stained with BLBP antibody. (Scale bar: 50 μm.) IZ, intermediate zone. (*D* and *F*) Summary of the thickness of the neonatal (E12.5) (*D*) and embryonic (P1) (*F*) cortices of mouse brains of both genotypes. **P < 0.01, one-way ANOVA and Tukey’s post hoc test.

As the laminar organization of the cortex depends on the normal radial process of RGCs (1, 24), the abnormal arrangement of cortical layers would be expected in *Ano1*^−/−^ mice if it is essential for the process maturation of RGCs (25). Thus, the cortices of *Ano1*^+/+^ and *Ano1*^−/−^ brains at P1 were stained with CUX1 and NOR1, markers of layers II–IV and layer V, respectively (26). *Ano1*^−/−^ mice are lethal within 2 wk after birth because of breathing difficulty due to abnormal development of trachealis muscle in the trachea (27). However, the gross shape of embryonic *Ano1*^−/−^ mice was not much different from that of *Ano1*^+/+^ mice, with similar body weight and shape of embryos in both genotypes (*SI Appendix*, Fig. S3 *A* and *B*). As shown in Fig. 5*A*, CUX1^+^ cells in the *Ano1*^+/+^ mice were localized only in layers II–IV and were rarely visible in other layers, whereas the cells from *Ano1*^−/−^ brains were distributed loosely throughout the entire cortical axis. Similarly, NOR1^+^ cells were localized mainly in layers V and VI in *Ano1*^+/+^ cortex (Fig. 5*B*). However, in the *Ano1*^*−/−*^ cortex, NOR1^+^ cells were also irregularly dispersed throughout the cortex. Thus, the layer-specific laminar organization in the cortex was disrupted in *Ano1*^−/−^ mouse brains.

In addition, because process extension in RGCs is important for maintaining normal cortical size (26), the cortical thickness of *Ano1*^+/+^, *Ano1*^+/−^, and *Ano1*^−/−^ mouse brains at E12.5 and P1 was measured with BLBP and Tuj-1 immunostaining. Cortical thickness was significantly decreased in embryonic *Ano1*^−/−^ mouse brains at E12.5 (*Ano1*^+/+^, 138.5 ± 3.1 μm; *Ano1*^+/−^, 124.0 ± 2.8 μm; *Ano1*^−/−^, 101.2 ± 2.6 μm; *n* = 12, 17, and 10, respectively; *P* < 0.01, *Ano1*^−/−^ vs. *Ano1*^+/+^, one-way ANOVA and Tukey’s post hoc test) (Fig. 5 *C* and *D*). Similarly, the thickness of the neuronal layer in the embryonic *Ano1*^−/−^ mouse brain (*Ano1*^+/+^, 21.5 ± 0.8 μm; *Ano1*^+/−^, 19.2 ± 0.8 μm; *Ano1*^−/−^, 16.8 ± 0.9 μm; *n* = 14, 15, and 8, respectively; *P* < 0.01, *Ano1*^−/−^ vs. *Ano1*^+/+^, one-way ANOVA and Tukey’s post hoc test) was also significantly decreased (Fig. 5 *C* and *D*). *Ano1*^−/−^ mice at P1 also exhibited a significant reduction in the thickness of the cortex (from VZ to cortical plate) (*Ano1*^+/+^, 888.0 ± 13.6 μm; *Ano1*^+/−^, 808.7 ± 14.9 μm; *Ano1*^−/−^, 786.7 ± 22.9 μm; *n* = 7, 8, and 7, respectively; *P* < 0.01, *Ano1*^−/−^ vs. *Ano1*^+/+^, one-way ANOVA and Tukey’s post hoc test) (Fig. 5 *E* and *F*). Because *Ano1* in the *Ano1*^−/−^ mice was ablated from the whole body, systemic factors other than cerebral ones would affect the change in cortical thickness. However, because the body weights of *Ano1*^−/−^ newborn pups at P1 were not different from those of WT littermates (*SI Appendix*, Fig. S3), systemic factors are not likely to affect the cortical thickness of *Ano1*^−/−^ brain.

RGCs are reported to undergo proliferative divisions at an early brain developmental stage (1, 2). Thus, we measured the number of proliferating cells in cultured NSCs by 5′-bromo-2′-deoxyuridine (BrdU) assay and in the cortex of *Ano1*^−/−^ brain at E14.5 by phospho-histone H3 assay. The number of BrdU^+^ cells was significantly lower in *Ano1* siRNA-transfected NSCs than in scrambled siRNA-transfected NSCs (*P* < 0.001, Student’s *t* test) (*SI Appendix*, Fig. S4*A*). Consistent with this, histone H3^+^ cells were much sparser in the cortex of *Ano1*^*−/−*^ brain than in the cortex of *Ano1*^*+/+*^ brain at E14.5 (*SI Appendix*, Fig. S4*B*). This result suggests that ANO1 also affects NSC proliferation.

### Brain-Derived Neurotrophic Factor Stimulates ANO1 in RGCs.

**Fig. 6.**
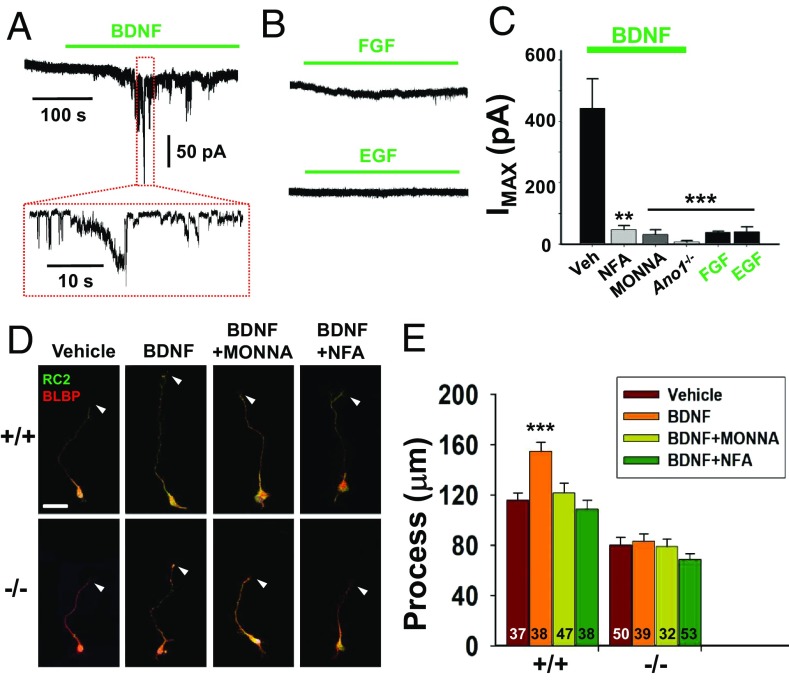
BDNF activates ANO1 in RGCs. (*A* and *B*) Representative traces of Cl^−^ current responses of cultured RGCs to BDNF (*A*) or trophic factors (*B*). Cells were treated with 100 ng/mL of BDNF, FGF, or EGF. Pipette and bath solutions contained 140 mM CsCl and 140 mM NMDG-Cl, respectively; *E*_hold_ = −80 mV. (*C*) Summary of average current amplitudes of RGCs activated by BDNF, FGF, EGF, or coapplication with 10 μM niflumic acid (NFA) and 30 μM MONNA. The average amplitudes of currents of RGCs isolated from *Ano1*^−/−^ brains are also shown. **P < 0.01, ***P < 0.001 vs. BDNF (Veh)-evoked currents, one-way ANOVA and Tukey’s post hoc test. (*D* and *E*) Representative images (*D*) and summary of process lengths (*E*) of RGCs treated with vehicle (Veh), BDNF, BDNF+NFA, or BDNF+MONNA in *Ano1*^+/+^ and *Ano1*^*−/−*^ RGCs for 3 d. Arrowheads indicate the tips of each process of RGCs. RGCs were stained with BLBP and RC2. (Scale bar: 20 μm.) ***P < 0.001, one-way ANOVA, Newman–Keuls multiple-comparison test.

ANO1 is known to be activated by G protein-coupled receptor-mediated ER-dependent cytosolic Ca^2+^ accumulation (11). Therefore, we postulated the ANO1-dependent process maturation of RGCs might have a Ca^2+^ source pathway. To investigate which upstream signals drive ANO1 to affect the process extension in RGCs, we treated various trophic factors known to stimulate NSC function (28, 29). Surprisingly, the application of brain-derived neurotrophic factor (BDNF) to cultured RGCs induced robust Cl^−^ currents that were blocked by the coapplication of 30 μM MONNA or 10 μM niflumic acid, blockers of ANO1 (Fig. 6 *A* and *C*) (23). However, other factors known to stimulate NSC growth, such as fibroblast growth factor (FGF) and epidermal growth factor (EGF), failed to activate ANO1-like Cl^−^ currents in RGCs (Fig. 6 *B* and *C*). The application of BDNF (100 ng/mL) evoked oscillatory Ca^2+^ spikes in RGCs (*SI Appendix*, Fig. S5). In addition, we also confirmed BDNF-mediated ANO1 activation with the coexpression of *TrkB* and *Ano1* in human embryonic kidney (HEK) 293T cells (*SI Appendix*, Fig. S6). More importantly, the application of 100 ng/mL BDNF to cultured RGCs induced process extension, which was reversed in RGCs isolated from *Ano1*^−/−^ mouse or when treated with MONNA or niflumic acid (Fig. 6 *D* and *E*). The coexpression of BDNF and ANO1 in the cortex was confirmed by RT-PCR or immunostaining (*SI Appendix*, Fig. S7 *A*–*C*). These results clearly suggest that ANO1 is essential for BDNF-induced process extension of RGCs in the developing brain.

## Discussion

RGCs are one type of NSCs and play a critical role in structural development of the brain. Two types of RGCs, apical and outer RGCs that are localized in the VZ and outer SVZ, respectively, play different roles in early brain development (30). Whereas the apical RGCs extend their processes to deliver and nurture immature neurons, the outer RGCs undergo self-renewing pathways and produce a large number of neural precursor cells (1, 5). These neural precursor cells migrate radially to construct the inside-out cortical structure in which neurons generate the deep infragranular layers (V and VI) first and the superficial layers (II–IV) later (30). In the present study, ANO1 appears to be an important regulator for RGC functions because its gene disruption or knockdown ameliorated the process extension of RGCs, whereas its activation promoted RGC process extension. In addition, *Ano1*^*−*/*−*^ brains showed an underdeveloped cortex with disrupted cortical layers and cortical thickness, further suggesting that ANO1 contributes to RGC function during cortical development.

RGCs are known to generate spontaneous Ca^2+^ transients autologously or from other RGCs. The Ca^2+^ transients are essential for RGC proliferation, neurogenesis (10), neurotransmitter phenotype, neurite outgrowth (9), and motility (31). Excitatory inputs to NSCs induce the Ca^2+^ transients, which depend on the activity of ion channels. Several channels are expressed in RGCs, including AMPA (32), voltage-gated sodium and potassium channels (33), voltage-gated calcium channel (34), and GABA_A_ receptors (7). Although GABA_A_ receptors are chloride channels, they generate depolarization on activation because of high intracellular Cl^−^ (35). The depolarization mediated by GABA_A_ receptors is known to regulate the proliferation and migration of NSCs (8–10). Because the depolarization induced by Cl^−^ efflux is essential for NSC activity, chloride channels other than the GABA_A_ receptors are of particular interest for NSC activity. The present study demonstrates that ANO1 expressed in RGCs is functionally active and promotes the maturation of RGC processes. Therefore, in addition to the GABA_A_ receptors, ANO1 as a Cl^−^ channel in NSCs can contribute to cortical development in the embryonic brain.

In RGCs, process extension is required for neuronal migration during cortical development (31, 36). Various soluble factors regulate the maturation of RGCs, including dystroglycan (37), glial growth factors (38), and glycogen synthase kinase 3 (39). The BDNF and its receptor TrkB are also expressed in NSCs (28, 40) and increase axonal process extension through the Wnt/β-catenin signaling pathway (41). Thus, BDNF might be linked functionally to ANO1 for RGC maturation. Indeed, BDNF is coexpressed with ANO1 in RGCs and evokes intracellular Ca^2+^ transients as well as MONNA-reversible Cl^−^ currents in RGCs (Fig. 6 *A* and *C* and *SI Appendix*, Figs. S5–S7). Thus, BDNF appears to contribute to RGC maturation in part via the ANO1 pathway.

The involvement of ANO2, another Ca^2+^-activated Cl^−^ channel in the Anoctamin family, in RGC function appears to be limited. ANO2 transcripts were rarely found in embryonic brains (*SI Appendix*, Fig. S2*C*). In addition, the genetic ablation of *Ano1*^*−*/*−*^ blocked the Ca^2+^-activated Cl^−^– or BDNF-induced currents in RGCs almost completely (Fig. 6*C*). Thus, the involvement of ANO2 in process maturation of RGCs may be minimal. However, when ANO2 and TrkB were overexpressed in HEK293T cells, BDNF generated an ANO2-dependent current (*SI Appendix*, Fig. S6). Thus, ANO2 may have a potential role in mediating neural functions other than in the developing brain (16).

Despite the finding of a role of ANO1 in RGC process maturation, the molecular mechanisms underlying this process remain unknown. One plausible theory comes from the involvement of intracellular Ca^2+^ in neurite extension in neurons. When a diffusible factor such as BDNF stimulates ANO1 via TrkB, the membrane becomes depolarized due to the efflux of Cl^−^. This depolarization stimulates the voltage-gated calcium channel, resulting in a large increase in intracellular Ca^2+^, which may regulate the process maturation via actin assembly (*SI Appendix*, Fig. S8). Numerous reports have suggested the involvement of Ca^2+^ in neurite growth or growth cone development; however, these reports are conflicting. Spontaneous Ca^2+^ spikes are observed in cultured neural precursor cells and are correlated with neurite length (9). In contrast, outgrowth of the growth cone is inversely correlated with the frequency of Ca^2+^ transients in *Xenopus* spinal neurons (42). Sustained Ca^2+^ increase slows axonal growth by stimulating calcineurin, which inhibits cytoskeletal assembly (43). Differences in cell type, cell lineage, or species may account for the conflicting results. Supporting the idea of actin assembly, ANO1 is associated with actin-binding regulatory proteins, such as ezrin, radixin, and moesin (44), the activation of which is responsible for cell polarity and motility (45). It is not clear how Ca^2+^ transients regulate the process maturation in RGCs. ANO1 may adjust the Ca^2+^ level locally and thereby regulate the process extension in RGCs (*SI Appendix*, Fig. S8).

In summary, ANO1 contributes to the process extension of RGCs during embryonic brain development. Because many brain disorders, such as autism, schizophrenia, and epilepsy, are related to defects in brain development, understanding the molecular mechanisms underlying the ANO1-dependent RGC functions may contribute to solving these mental disorders.

## Materials and Methods

### Live Cell Imaging.

Scrambled or *Ano1* siRNA-transfected RGCs were plated at a density of 100,000 cells/cm^2^ on a 24-well plate. The cells were incubated in a humidified live-cell imaging chamber with 5% CO_2_, 90% N_2_, and 5% O_2_ and then photographed every 1 h for 80 h in an IncuCyte ZOOM automated microscope system (Essen BioScience).

### Generation of ANO1 KO Mice.

*Ano1*^*+/−*^ C57/BL6 mice were provided by Brian D. Harfe. *Ano1*^*+/+*^, *Ano1*^*+/−*^, and *Ano1*^*−/−*^ embryonic or neonatal mice were obtained after crossing the heterozygotes.

### Electrophysiology.

Whole-cell currents were recorded at a holding potential of −80 mV using a voltage-clamp technique with an Axopatch 200B amplifier (Molecular Devices). The pipette solution contained (in mM) 136 CsCl, 2 MgCl_2_, 10 Hepes, 10 d-mannitol, 2 Mg-ATP, and 0.2 Na-GTP (pH adjusted to 7.2). The extracellular solution contained 140 *N*-methyl-d-glucamine-Cl (NMDG-Cl), 2 MgCl_2_, 10 d-mannitol, and 10 Hepes. To record the intracellular Ca^2+^-activated Cl^−^ current, 1 μM CaCl_2_ (Sigma-Aldrich) was added to the pipette solution. For the voltage pulse experiment, voltage steps were applied 1 s apart between −100 mV and +100 mV in 20-mV increments at a holding potential of −60 mV. For embryonic brain slice preparation and ex vivo current recording, sagittal 400-µm-thick slices of E13.5 or E14.5 embryonic mouse head were prepared using a vibratome in ice-cold artificial cerebrospinal fluid. RGCs of the VZ were selected for whole-cell patch clamp recording (resistance of patch pipettes, 7–10 MΩ). Whole-cell currents were recorded using a voltage-clamp technique with an Axopatch 700B amplifier, and then digitized using Digidata 1440 (Molecular Devices). The sampling rate of the current was 10 kHz, and data were analyzed using pCLAMP version 10 (Molecular Devices).

### Statistical Analysis.

Data are presented as mean ± SEM. Statistical significance was analyzed using Student’s *t* test. For comparison of multiple means, one-way ANOVA followed by Tukey’s or Newman–Keuls post hoc test was used. *P* < 0.05 is considered significant.

## Supplementary Material

Supplementary File
