# Supplementary material for: ANO1/TMEM16A regulates process maturation in radial glial cells in the developing brain
Source: Proc Natl Acad Sci U S A. 2019 May 30;116(25):12494–9. doi: 10.1073/pnas.1901067116 (PMC6589654; doi:10.1073/pnas.1901067116)
Supplement: Supplementary File [file pnas.1901067116.sapp.pdf]

PNAS
www.pnas.org

Supplementary Information for

**ANO1/TMEM16A Regulates Process Maturation in Radial Glial Cells in the Developing Brain**

Gyu-Sang Hong[1+], Sung Hoon Lee[3+], Byeongjun Lee[1], Jae Hyouk Choi[2], Soo-Jin Oh[1], Yongwoo Jang[4], Eun Mi Hwang[1,2], Hyungsup Kim[1], Jooyoung Jung[1], In-Beom Kim[5], and Uhtaek Oh[1*]

[+] Equal contribution

* Correspondence to U Oh (utoh@kist.re.kr tel: 82-2-958-7033)

**This PDF file includes:**

Supplementary text
Figs. S1 to S8



www.pnas.org/cgi/doi/10.1073/pnas.1901067116

## Supplementary Information Text

## Supplementary methods

### Neurosphere-derived primary RG culture

All animal experiments were performed using protocols approved by the Seoul National University Institutional Animal Care and Use Committees. Neurospheres were prepared from the embryonic cortex of E12.5 C57BL/6 mice (DBL, Chungbuk, Korea). The embryonic cortices were dissociated into single cells by repeated mechanical trituration and passed through a 40 μm strainer (BD Bioscience, Franklin Lakes, NJ). The dissociated single cells were grown with Dulbecco's modified Eagle's medium (DMEM)/F12 (11320033, Gibco) supplemented with B27 (17504044, Invitrogen), 20 ng/ml of EGF (01107, Millipore) and 10 ng/mL of bFGF (13256029, Invitrogen) in a 5% $CO_2$, 90% $N_2$, and 5% $O_2$ humidified incubator. These cells were grown into floating neurospheres for 5-7 days. To make the differentiating condition, these neurospheres were dissociated into single cells with TrypLE™ (12605010, Invitrogen) and plated on a poly-L-lysine (P9155, Sigma)-coated plate with DMEM/F12 media containing 2% penicillin/streptomycin without the growth factors. Three days after plating, the cells were fixed with 4% paraformaldehyde and immunostained with RC2 (MAB5740, Millipore) and BLBP antibodies (ab32423, Abcam) to check the population of RGCs. For electrophysiology, the cells were chosen by morphology showing oval soma with monopolar or bipolar processes, similar to the RC2 and BLBP positive cells.

### Immunofluorescence Staining

Neurosphere-derived RGCs or directly isolated RGCs from *Ano1*$^{-/-}$ or *Ano1*$^{+/+}$ mice were plated on coverslips coated with 50 μg/mL poly-L-lysine (Sigma-Aldrich) and fixed with 4% paraformaldehyde in 3 days after plating at 4°C for 20 min. The fixed cells were permeabilized with 0.1% Triton X-100 at room temperature for 15 min and blocked with 1% bovine serum albumin in phosphate-buffered saline (PBS). The cells were incubated overnight at 4°C with primary antibody diluted with a blocking buffer. After washing with PBS, the cells were incubated with secondary antibodies conjugated with either Alexa Fluor® 488 dye (1:400; Invitrogen) or Alexa Fluor® 555 dye (1:400; Invitrogen) at room temperature for 90 min. After washing with PBS, the coverslips were mounted with

Vectashield (Vector, Burlingame, CA) and imaged using a confocal microscope (LSM 700, Carl Zeiss, Jena, Germany).

For immunostaining mouse brain, brains were separated from embryonic or postnatal mice deeply anesthetized with $CO_2$ gas. The isolated brains were fixed with 4% paraformaldehyde. The brains were embedded in paraffin and cut into 3 μm sections using a vibratome. After deparaffinization, rehydration, and antigen retrieval by microwave heating at boiling in a citrate buffer (pH 6.0), the sections were blocked with PBS containing 5% normal goat serum and 0.3% Triton™ X-100 for 1 h at room temperature and incubated overnight at 4°C with primary antibodies diluted in PBS containing 1% bovine serum albumin and 0.3% Triton X-100. After washing three times, sections were incubated with secondary antibodies. Images were captured with a Zeiss LSM 700 confocal microscope.

For immunostaining of cortical layers, the brains were isolated and fixed in 4% paraformaldehyde. Sections were washed and incubated with blocking buffer (5% (wt/vol) goat serum or 2% bovine serum albumin in PBS with 0.2% Triton-X) for 1 hour. CUX1 (SC-13024, Santa Cruz, 1:20) and NOR1 (Ab94507, Abcam, 1:200) antibody was incubated with samples in the blocking buffer overnight at 4°C. Samples were then washed and incubated with Alexa Fluor® 488 dye (1:500; Invitrogen) at room temperature for 1 hour. Nucleus was stained using Hoechst 33342 for 10 min (H3570, Thermofisher Scientific, 1:2000) The sections were imaged with a confocal microscope (LSM700, Carl Zeiss).

**Transfection**

The RGCs were transfected with *Ano1* or scrambled siRNA (Bioneer, Korea), pEGFP-N1, or ANO1-pEGFP-N1 using a Neon™ Transfection System electroporation (Invitrogen) according to the manufacturer's guide. RGCs at a density of 100,000 cells/$cm^2$ were plated for immunostaining or live-cell imaging.

**Western blot**

A sample of 20-40 μg of protein was collected from neurosphere-derived RGCs with a RIPA buffer (GenDEPOT Barker, TX). The proteins were separated using 8% SDS-polyacrylamide gel electrophoresis and transferred onto PVDF membranes (Whatman, Hahnestraβe, Dassel, Germany). The membranes were blocked with 5% skim milk in Tris-buffered saline (TBS) for 1 h at room temperature, then incubated overnight at 4°C

with an antibody against ANO1 (1:2000, Abfrontier, Korea), Nestin (1:5000; Millipore), Tuj-1 (1:10,000, Covance, Prinston, NJ), GFAP (1:5000; Dako, Carpinteria, CA), Synapsin 1 (1:10,000, BD bioscience, Franklin Lakes, NJ), GAD67 (1:5,000; Millipore), and β-actin (1:20,000, Sigma). They were then washed three times for 10 min each with TBS-Tween (0.2% Tween-20) and incubated with horseradish peroxidase-conjugated secondary antibody for 90 to 120 min at room temperature. The membranes were developed with enhanced chemiluminescence solution (iNtRON, Seoul, Korea). Protein bands were detected with ImageQuant LAS-4000 (GE Healthcare).

## AUTHOR CONTRIBUTIONS

GSH and SL designed the experiments, GSH performed immunohistochemistry and electrophysiology, SL carried out RGC culture, immunohistochemistry, RGC process extension, BL and HK maintained the knockout animals, YJ, EH, and JJ undertook the molecular work, IBK worked on immunohistochemistry, GSH, JC and SJO performed *ex vivo* electrophysiology, and OU supervised the experiments and wrote the manuscript.

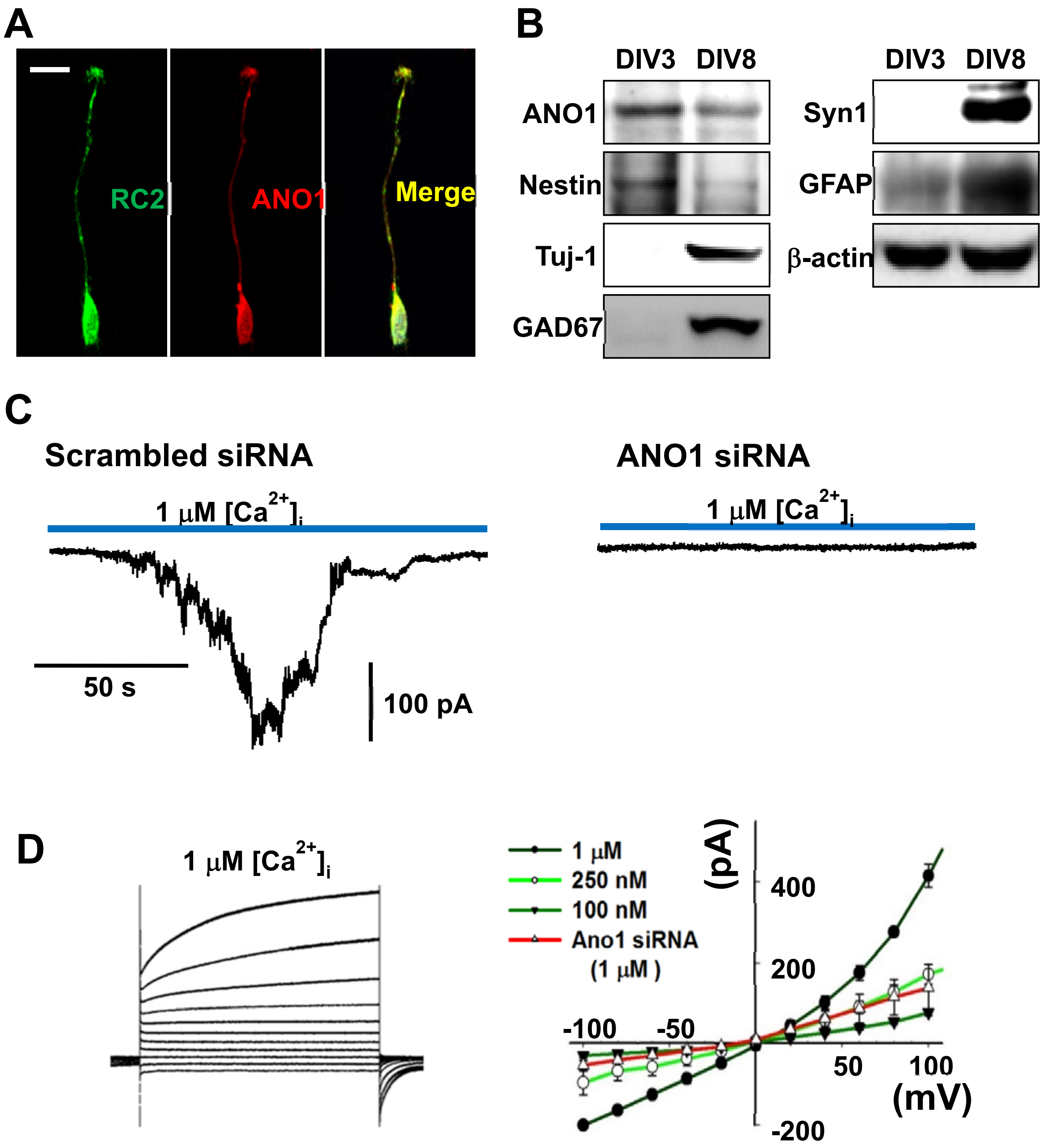
A
RC2
ANO1
Merge
B
DIV3 DIV8
ANO1
Nestin
Tuj-1
GAD67
DIV3 DIV8
Syn1
GFAP
β-actin
C
Scrambled siRNA
1 μM [Ca2+]i
50 s
100 pA
ANO1 siRNA
1 μM [Ca2+]i
D
1 μM [Ca2+]i
1 μM
250 nM
100 nM
Ano1 siRNA
(1 μM )
(pA)
400
200
-200
-100
-50
50
100
(mV)

**Fig. S1. Functional expression of ANO1 in cultured RGCs.**

(*A*) Immunofluorescence images of cultured RGCs stained with ANO1 and RC2 antibodies. RGCs were isolated from neurospheres and cultured for 3 days after plating. Scale bar : 20 μm.

(*B*) Expression of ANO1 in cultured RGCs determined by immunoblotting. Tuj-1, glutamate decarboxylase 67 (GAD67), and synapsin 1 (Syn1) were used as neuronal markers. Nestin and glial fibrillary acidic protein (GFAP) were used as NSC and glial cell markers, respectively. β-actin was used as a control.

(*C*) Representative trace of $Ca^{2+}$-activated $Cl^-$ currents in cultured RGCs transfected with scrambled (left trace) or *Ano1* siRNA (right trace). *Ano1* siRNA was conjugated with Cy3 to visualize the siRNA-transfected cells. To record $Ca^{2+}$-activated $Cl^-$ currents, the pipette contained 1 μM $Ca^{2+}$ in 140 mM CsCl solution. The bath solution contained 140 mM N-methyl-D-glucamine (NMDG)-Cl. $E_{hold}$ = -80 mV.

(*D*) Representative of traces of $Ca^{2+}$-activated $Cl^-$ currents in cultured RGCs in response to voltage steps (-100 to +100 mV, 20 mV increment). The pipette contained 1 μM $Ca^{2+}$ (left panel). Current-voltage relationships of the $Ca^{2+}$-activated $Cl^-$ currents at different $Ca^{2+}$ concentrations (100 nM, 250 nM and 1 μM) (right panel). Transfection of *Ano1* siRNA into the RGCs abolished the $Ca^{2+}$-activated chloride currents (n = 5–8).

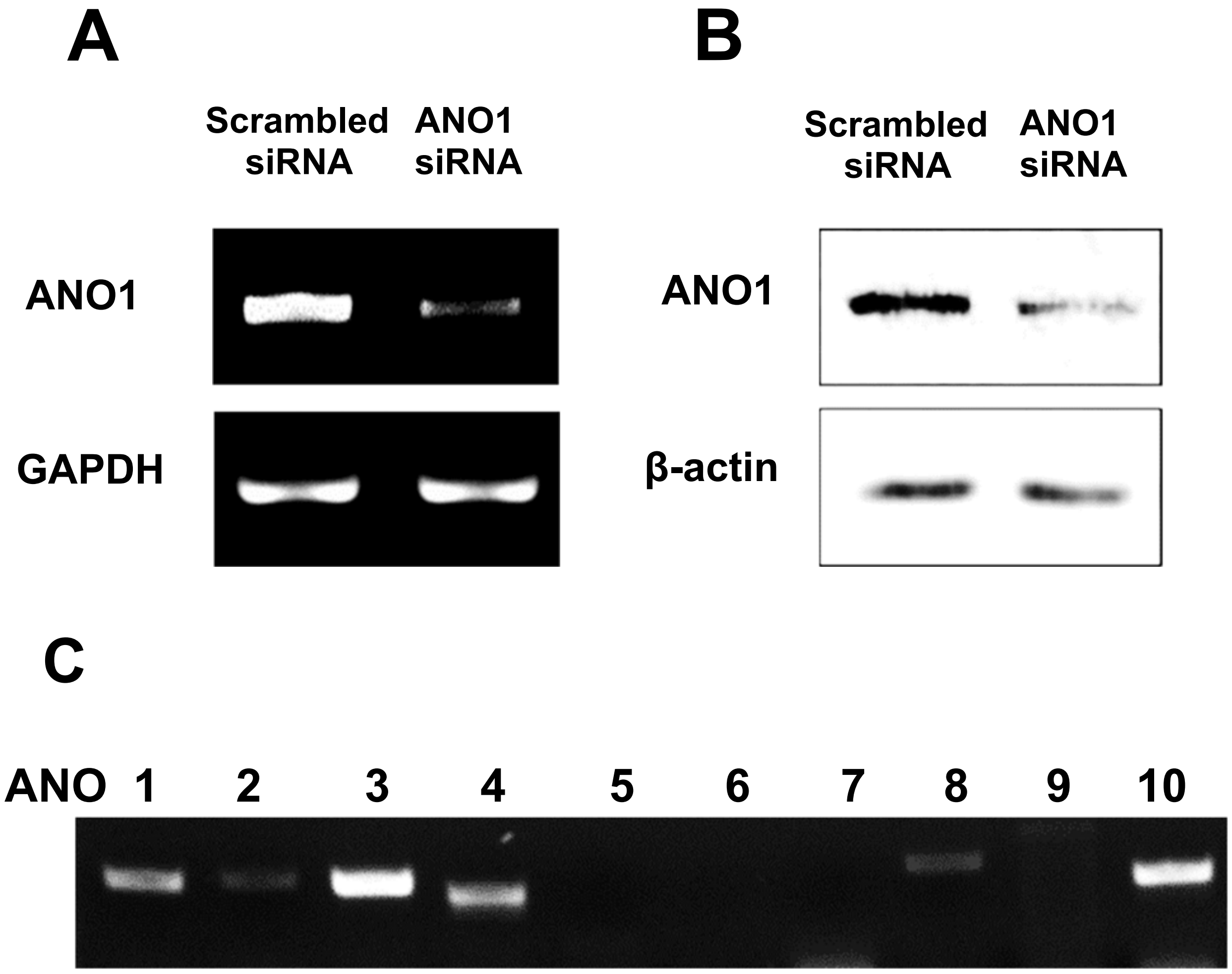


**Fig. S2. Ano1 transcripts and proteins and the expression of *Ano* family genes in cultured RGCs.**

(*A*, *B*) The mRNA (*A*) and protein (*B*) levels of ANO1 in cultured RGCs were assayed with RT-PCR and immunoblotting. The RGCs were transfected with *Ano1* or scrambled siRNA.

(*C*) The PCR products of *Ano* family genes in cultured RGCs.

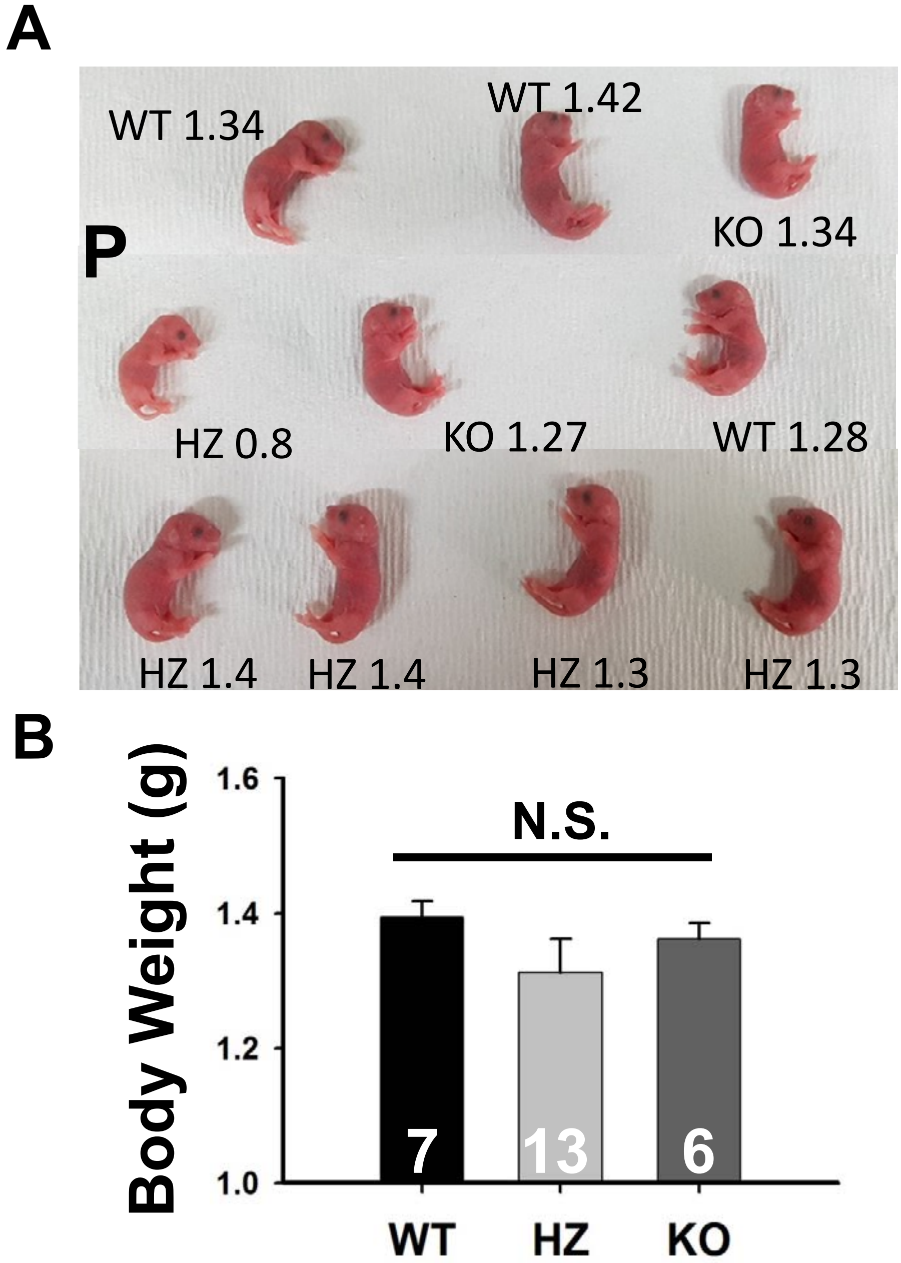


**Fig. S3. Body weights of *Ano1*$^{+/+}$, *Ano1*$^{+/-}$ and *Ano1*$^{-/-}$ neonatal (P1) mice.**

(*A*) Representative pictures of *Ano1*$^{+/+}$ (WT), *Ano1*$^{+/-}$ (HZ) and *Ano1*$^{-/-}$ (KO) P1 littermates. The body weight of each pup is shown along with its genotype.

(*B*) Summary of the body weights of *Ano1*$^{+/+}$, *Ano1*$^{+/-}$ and *Ano1*$^{-/-}$ P1 littermates. N.S. non-significant.

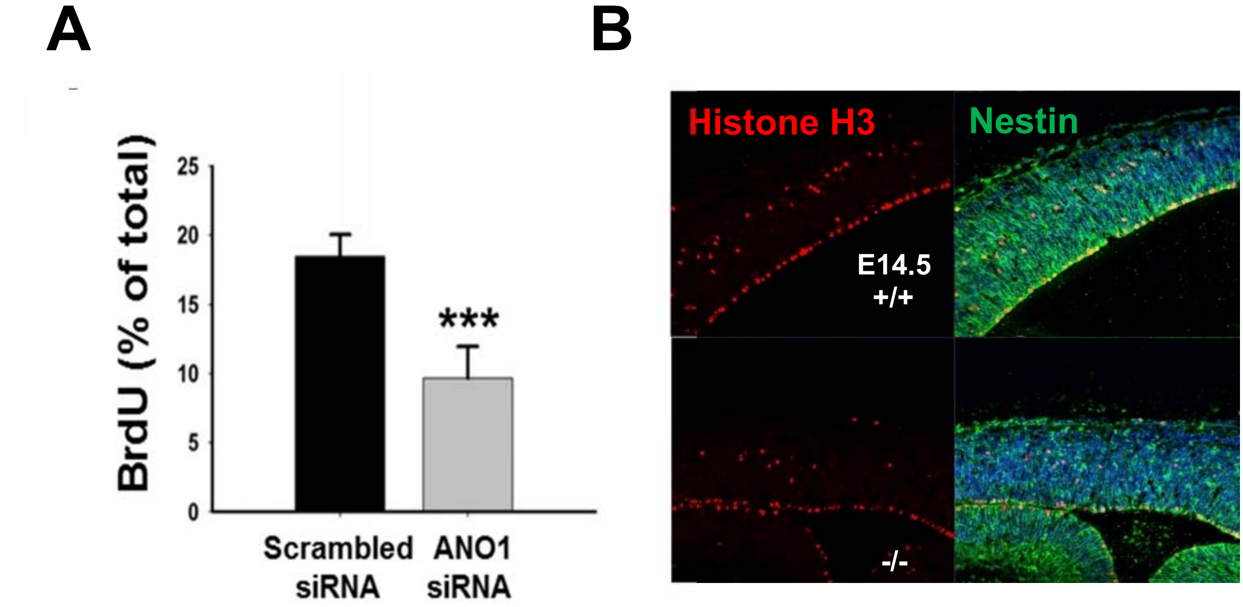


**Fig. S4. The proliferation of cultured NSCs by *Ano1* knock-down and in the cortex of *Ano1*$^{-/-}$ embryonic brains.**

(A) BrdU-positive cells were measured in cultured RGCs transfected with *Ano1*-siRNA or scrambled siRNA. BrdU+ cells were counted as a percentage of the total cells.

(B) Histone H3-postive cells were immunostained with Nestin antibody in E14.5 embryonic brain sections of cortices from *Ano1*$^{+/+}$ (WT) and *Ano1*$^{-/-}$ (KO). Note that Histone H3-positive cells were much sparser in *Ano1*$^{-/-}$ embryonic brain than in *Ano1*$^{+/+}$ brain.

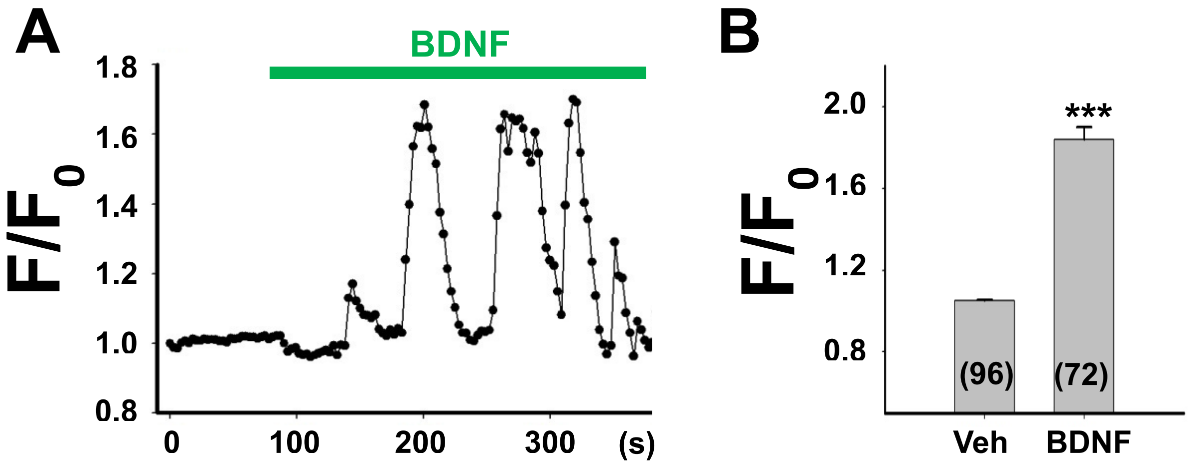


**Fig. S5. A representative trace (A) and summary (B) of BDNF-induced $Ca^{2+}$ spikes in cultured RGCs from the wild-type brains.** RGCs were pretreated with Fluo-3 AM before the $Ca^{2+}$ imaging. ****P* < 0.001, Student's t-test.

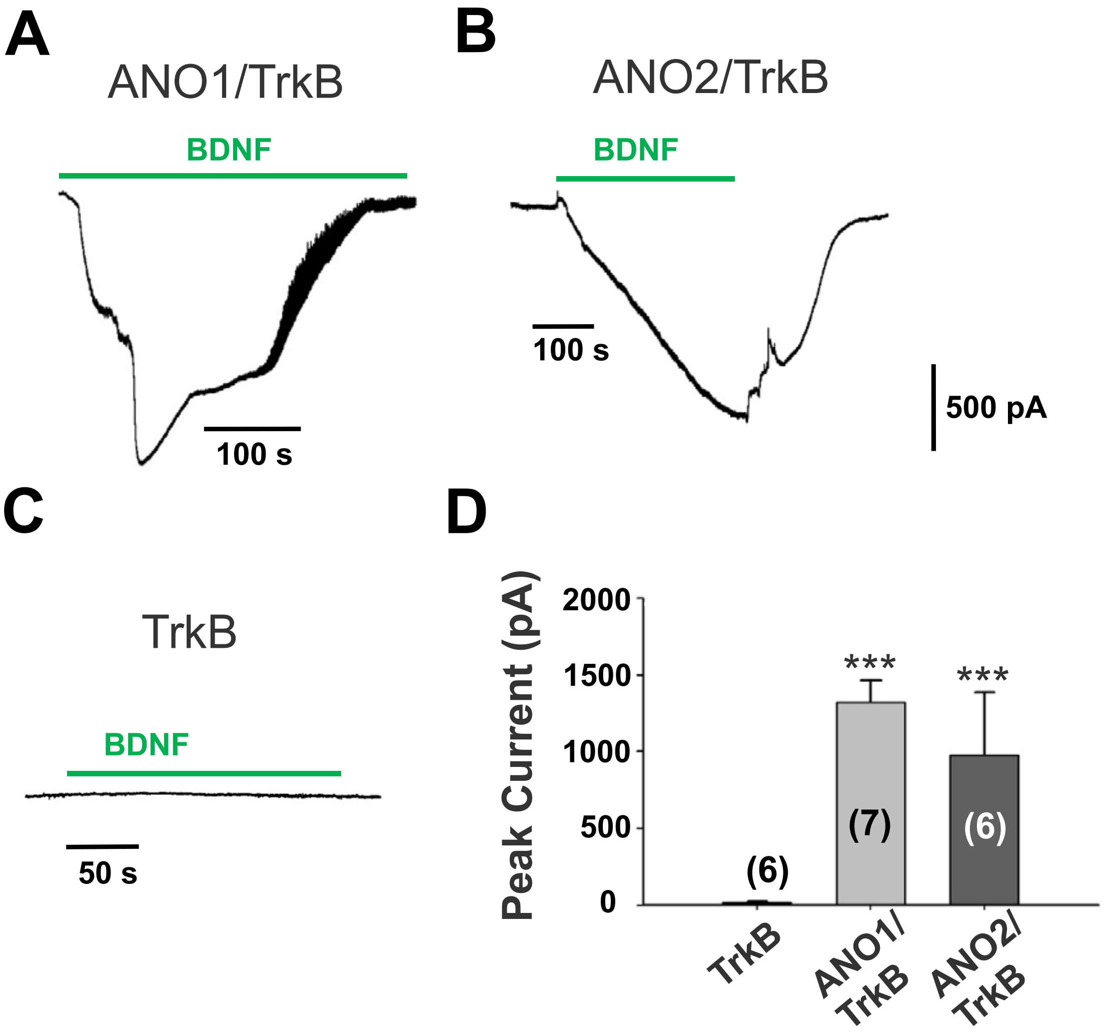


**Fig. S6. Activation of ANO1 and 2 by BDNF in HEK293T cells.**

(A-C) Representative traces of BDNF-induced $Cl^-$ currents in HEK293T cells transfected with *Ano1* + *TrkB* (A), *Ano2* + *TrkB* (B), and *TrkB* only (C).

(D) Summary of BDNF-induced $Cl^-$ currents in HEK293T cells transfected with *Ano1* + *TrkB*, *Ano2* + *TrkB*, and *TrkB* only.

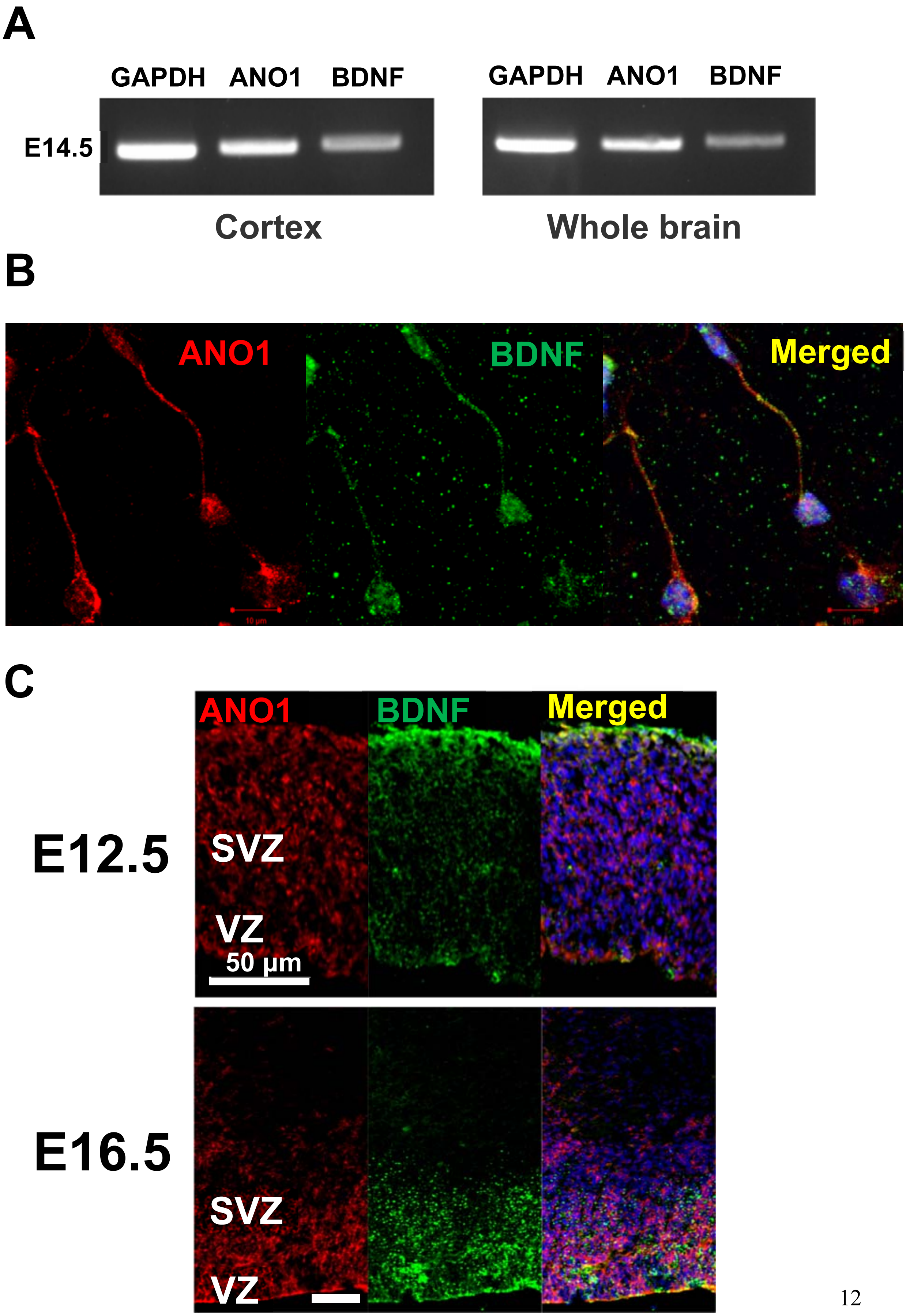
A
GAPDH
ANO1
BDNF
GAPDH
ANO1
BDNF
E14.5
Cortex
Whole brain
B
ANO1
BDNF
Merged
C
ANO1
BDNF
Merged
E12.5
SVZ
VZ
50 µm
E16.5
SVZ
VZ

**Fig. S7. Expression of ANO1 and BDNF in the embryonic cortex and whole brain (E14.5).**

(A) Expressions of ANO1 and BDNF were determined in the freshly-isolated cortex and whole brain from E14.5 mouse with RT-PCR.

(B) Co-localization of ANO1 and BDNF in cultured RGCs shown in immunofluorescent images.

(C) Immunofluorescent images stained with ANO1 and BDNF antibodies in E12.5 and E16.5 embryonic cortices.

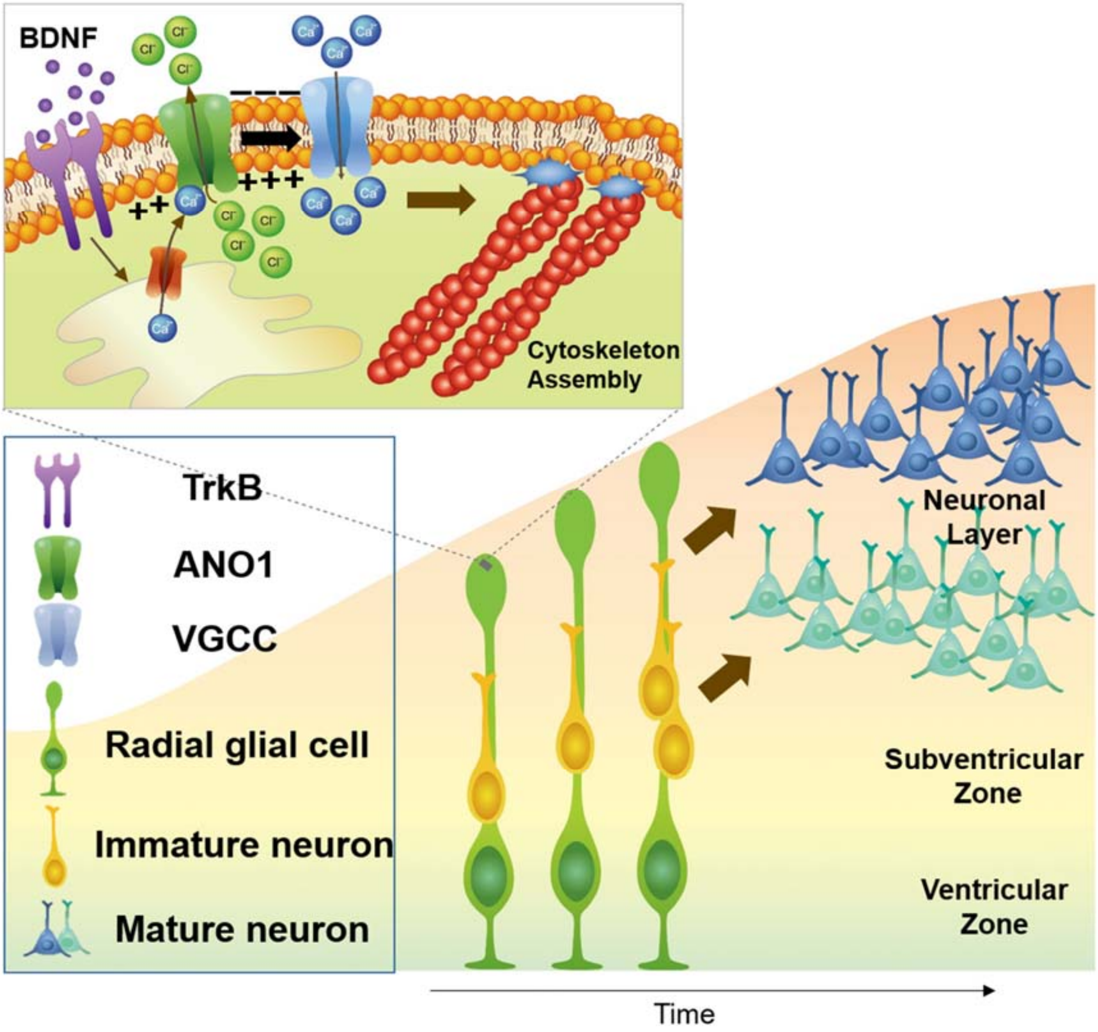


**Fig. S8. Schematic diagram showing the molecular mechanism underlying the role of ANO1 in process maturation of RGCs.**
